# Spina Ventosa of the Left Ring Finger With Tuberculous Pyomyositis in the Forearm Muscles in a Young Immunocompetent Indian Male With No Pulmonary Involvement: A First Report of Its Type

**DOI:** 10.7759/cureus.66823

**Published:** 2024-08-13

**Authors:** Sankalp Yadav, Madhan Jeyaraman, Gautam Rawal, Naveen Jeyaraman

**Affiliations:** 1 Medicine, Shri Madan Lal Khurana Chest Clinic, New Delhi, IND; 2 Orthopedics, South Texas Orthopedic Research Institute, Laredo, USA; 3 Clinical Research, Virginia Tech India, Dr. MGR Educational and Research Institute, Chennai, IND; 4 Orthopedics, ACS Medical College and Hospital, Dr. MGR Educational and Research Institute, Chennai, IND; 5 Respiratory Medical Critical Care, Max Super Speciality Hospital, Saket, IND

**Keywords:** cartridge-based nucleic acid amplification test (cbnaat), mycobacterium tuberculosis (mtb), tb, phalanx, ring finger, spina ventosa

## Abstract

Tuberculous infection of the extrapulmonary sites, especially the small bones, is a seldom reported entity even in endemic countries. Moreover, simultaneous involvement of the forearm muscles is a very rare presentation with no such case reported showing concurrent involvement of the two sites. The diagnosis is challenging due to the paucibacillary nature of the disease, a lack of awareness among primary clinicians, and ambiguity in clinical features with other musculoskeletal disorders, especially when there is no pulmonary involvement. Herein, we present a first-of-its-type case of spina ventosa of the left ring finger with a tuberculous abscess in the forearm in a 15-year-old Indian male with no pulmonary seeding. The diagnosis was achieved through a detailed diagnostic workup, which resulted in the detection of *Mycobacterium tuberculosis*. He was initiated on antituberculous treatment with a remarkable improvement.

## Introduction

Tuberculous dactylitis, or spina ventosa, is a rare type (2-4% of total skeletal tuberculosis) of tuberculosis affecting the small bones in the hands or feet [1]. It typically occurs due to the spread of *Mycobacterium tuberculosis* through the bloodstream from the lungs. This condition is uncommon in individuals older than six years of age as it usually develops before the epiphyseal centers in bones form. Symptoms of tuberculous dactylitis typically appear one to three years after the initial infection [2].

Additionally, pyomyositis of the forearm muscles is the least common site of extraspinal musculoskeletal tuberculosis. The musculoskeletal system's involvement by the tuberculous process is highly unusual, and cases of both immunocompromised and immunocompetent persons have been reported to have tuberculous pyomyositis [3].

Further, extrapulmonary tuberculosis constitutes about 10-15% of total tuberculosis cases [4]. Moreover, musculoskeletal involvement occurs in only 1-5% of tuberculosis cases, constituting approximately 10-18% of all extrapulmonary tuberculosis instances [1,5]. Despite being endemic in some regions, there is limited literature available on tuberculosis affecting the small bones of the hand [5]. Concurrent involvement of abscess in the forearm muscles is exceedingly rare. Herein, a case of spina ventosa of the left ring finger with tubercular abscess in the forearm in a young Indian male with no pulmonary seeding is presented. To the best knowledge of the authors, such a presentation has never been reported in this age group.

## Case presentation

A 15-year-old non-diabetic Indian male from a middle-income family came to the outpatient department with complaints of painful swelling in his left ring finger and left forearm for 45 days. The swellings began slowly over the course of four months and were eventually accompanied by pain. His everyday activities were affected by the pain, which started out mildly. After using a nonsteroidal anti-inflammatory medication that he could buy over the counter, his discomfort temporarily decreased.

There was no past history of fever, coughing, weight loss, or any other constitutional symptoms associated with tuberculosis. He was a student and had never smoked. Neither he nor any of his acquaintances had ever suffered from tuberculosis. Moreover, there was no past record of trauma, falls, or stays at refugee camps or night shelters. There was no history of child abuse.

A general assessment revealed that the patient's hemodynamics were stable. He did not exhibit any obvious signs of pallor, icterus, clubbing, cyanosis, or pretibial edema. He was of a slender frame (body mass index 18.0 kg/m²). His systemic review showed nothing noteworthy.

**Figure 1:**
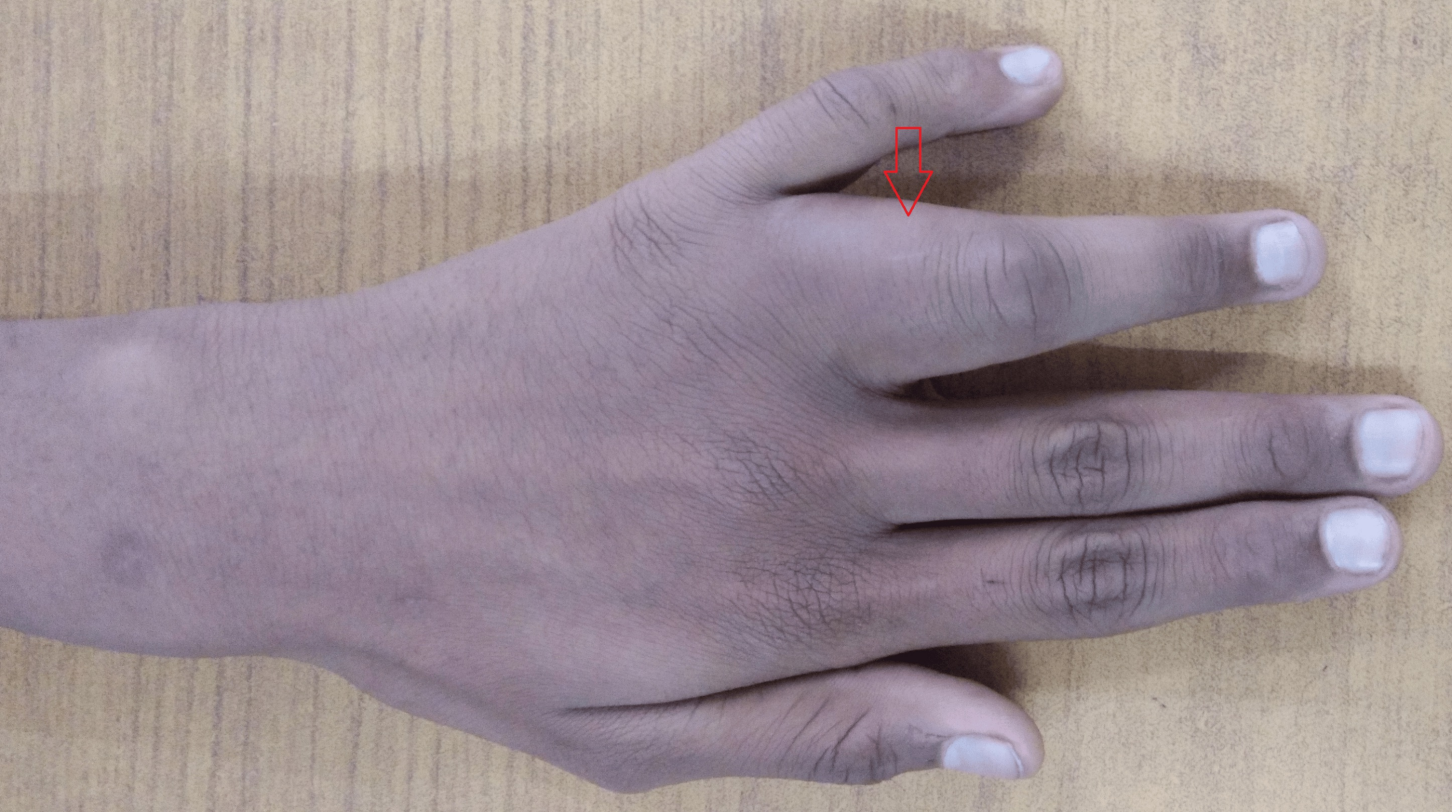
Gross image of the left ring finger showing the swelling.

On local examination, the proximal phalanx of the left ring finger was noticeably enlarged, tender to touch, hard, and fusiform, with mild erythema in the adjoining skin but no local rise in temperature (Figure 1).

**Figure 2:**
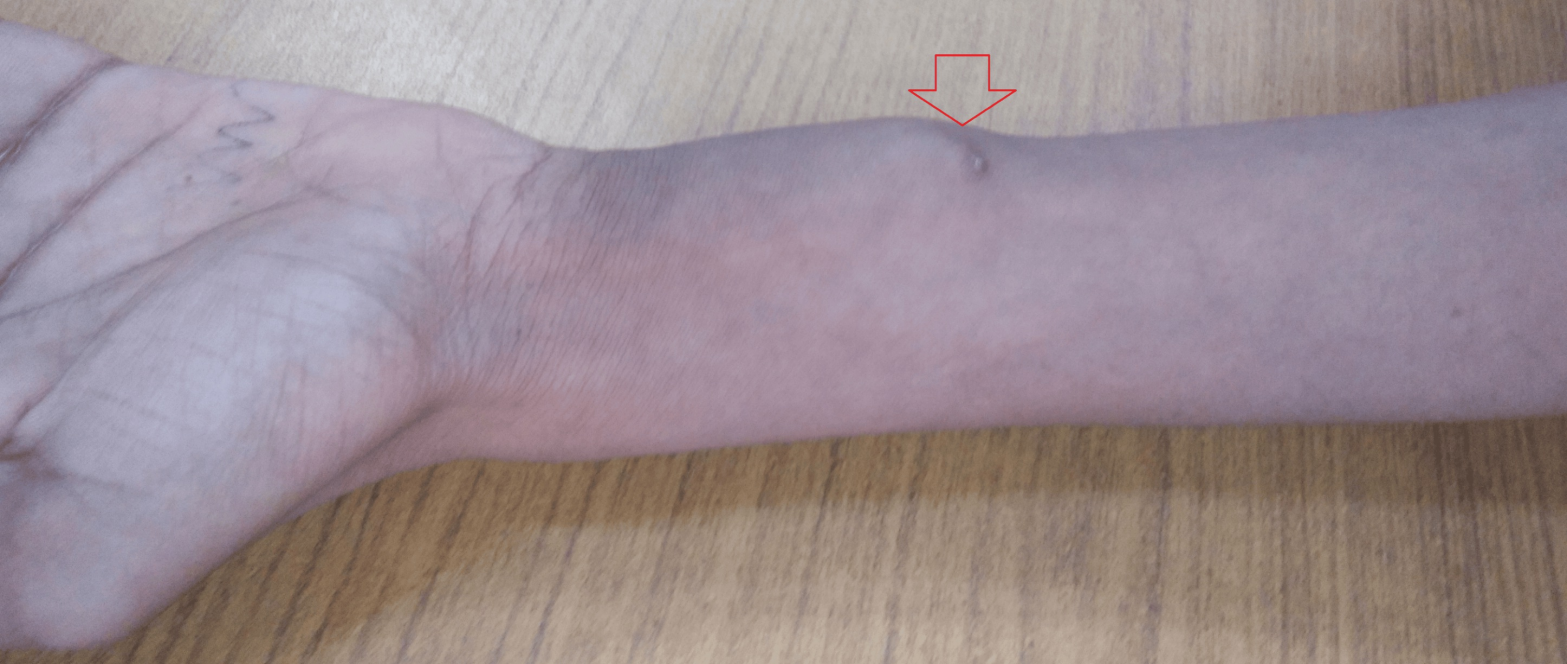
Gross image of the distal left forearm showing the swelling.

Further swelling was noted on the volar aspect of the distal left forearm. It was about 3 x 4 cm fixed with mild tenderness and raised local temperature (Figure 2).

While considering the possibility of fungal, syphilitic, enchondroma, brucellosis, and pyogenic osteomyelitis, a preliminary diagnosis of tuberculous dactylitis was made. It was recommended that he get routine radiographs of his left hand and chest as well as routine serological testing. A chest radiograph showing no evidence of pulmonary tuberculosis was obtained.

**Figure 3:**
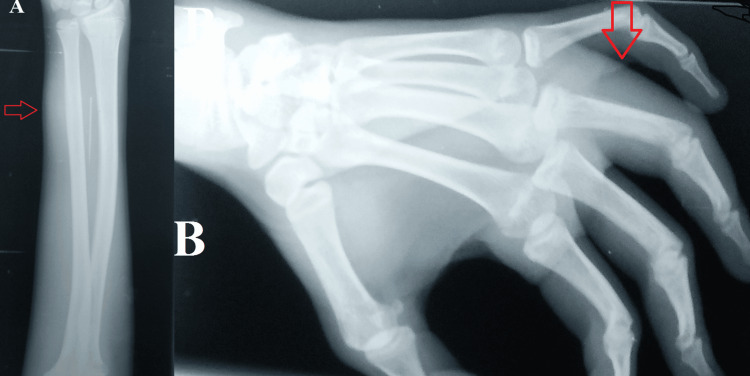
The left hand's plain radiographs. (A) Anteroposterior view showing the soft tissue swelling. (B) Oblique view showing the swollen ring finger.

According to the laboratory results, there was an elevated erythrocyte sedimentation rate of 49 mm in the first hour and 11.0 g/dL of hemoglobin. His hepatitis panel (A, B, and C), HIV, and laboratory testing for venereal disease research were all negative. In addition, the results of the brucella antibody titer, leukocyte count, absolute lymphocyte and neutrophil count, and absolute eosinophil count were unremarkable. His Mantoux test result was significantly positive (35 x 20 mm), but the rheumatoid factor was negative. The left hand's plain radiographs revealed soft tissue swelling, modest periosteal response, and cortical erosion in the distal portion of the fourth proximal phalanx, which combined to provide the appearance of spina ventosa (Figure 3).

**Figure 4:**
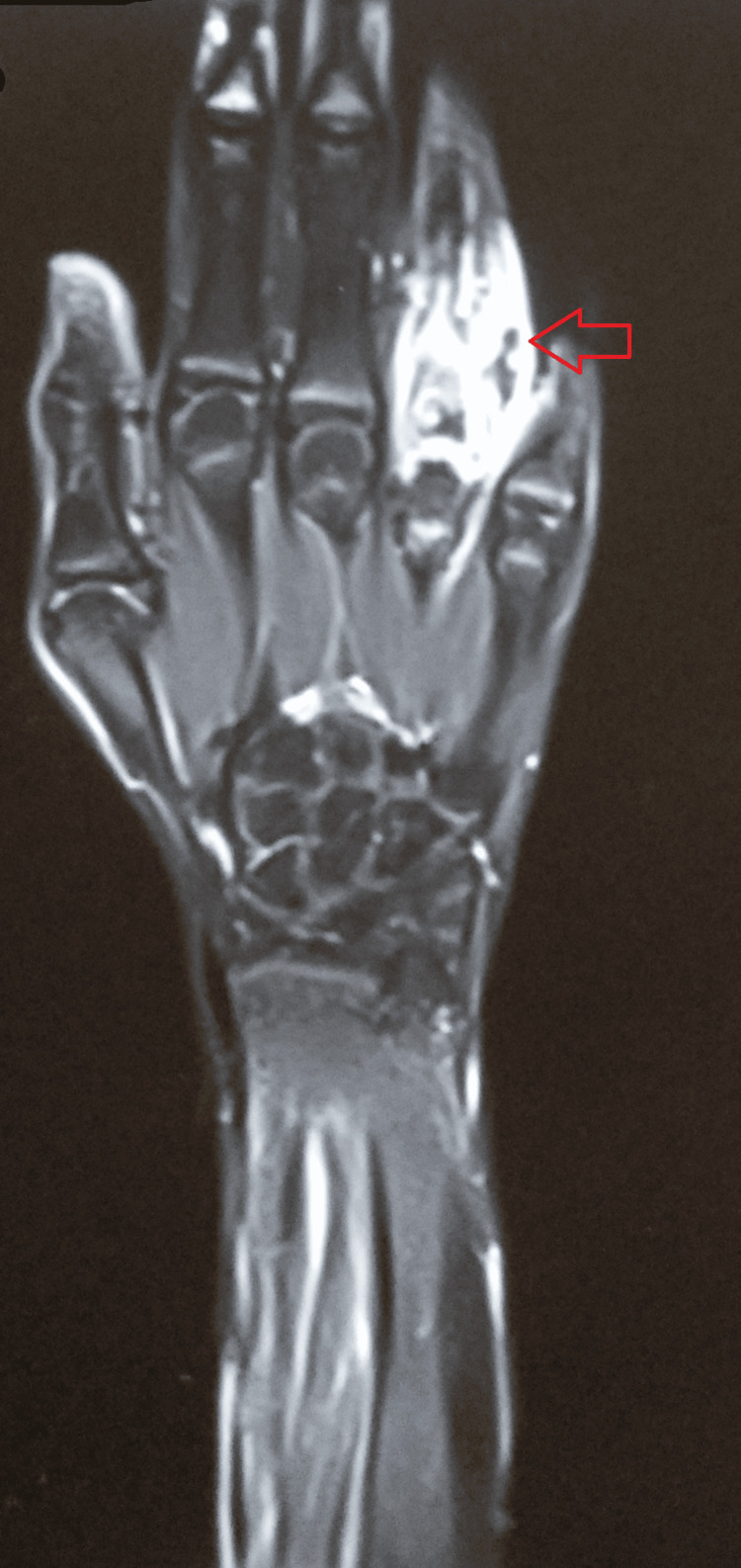
CEMRI of the left upper extremity revealing an altered signal intensity involving the proximal phalanx of the fourth digit. CEMRI: Contrast-enhanced magnetic resonance imaging

**Figure 5:**
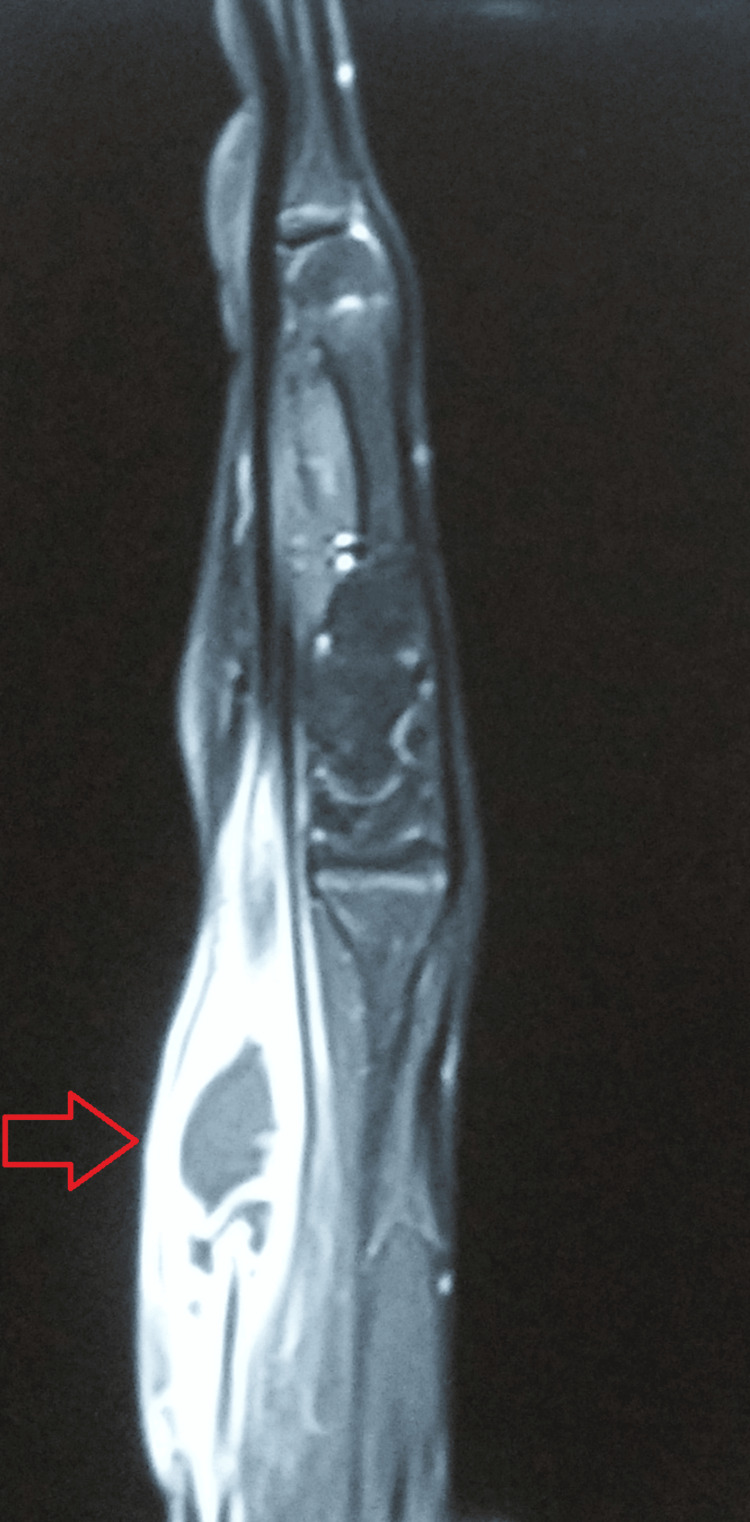
CEMRI showing peripherally enhanced collection in the distal forearm in the volar aspect with significant soft tissue edema. CEMRI: Contrast-enhanced magnetic resonance imaging

Contrast-enhanced magnetic resonance imaging of the left upper extremity revealed that there was an altered signal intensity involving the proximal phalanx of the fourth digit. There was associated cortical irregularity and erosions, as well as surrounding lamellated periosteal reactions. There was a thick and peripherally enhancing loculated collection in the surrounding soft tissue measuring 2.7 x 2.0 x 4.5 cm. The subcutaneous planes also appeared to be thickened and inflamed. There was another peripherally enhanced collection measuring 2.5 x 2.1 x 3.9 cm in the distal forearm in the volar aspect with significant soft tissue edema (Figures 4-5).

A fine-needle aspiration of the pus was done from the left forearm swelling which was suggestive of tuberculosis with the presence of sheets of viable and degenerate polymorphs and phagocytic histiocytes in the background of necrotic material. Staining for acid-fast bacilli was positive. Following an ultrasound-guided biopsy from the left ring finger and left forearm swelling, granulomatous inflammation and Langhans giant cells affecting the dermis and subcutaneous fat with a necrotic background were seen. The samples tested negative for bacteria, fungus, and mycobacteria using Gram staining and culture. Samples were also sent for testing using cartridge-based nucleic acid amplification. *Mycobacterium tuberculosis *(medium) was detected in both samples, and neither sample exhibited rifampicin resistance. With a final diagnosis of spina ventosa of the left ring finger with tuberculous pyomyositis of the distal left forearm, he was consequently recommended a 12-month course of fixed-dose combination of anti-tubercular chemotherapy with four drugs, i.e., rifampicin, pyrazinamide, ethambutol, and isoniazid, for 56 days, followed by three drugs (rifampicin, ethambutol, and isoniazid) in the in the continuation phase for 10 months.

**Figure 6:**
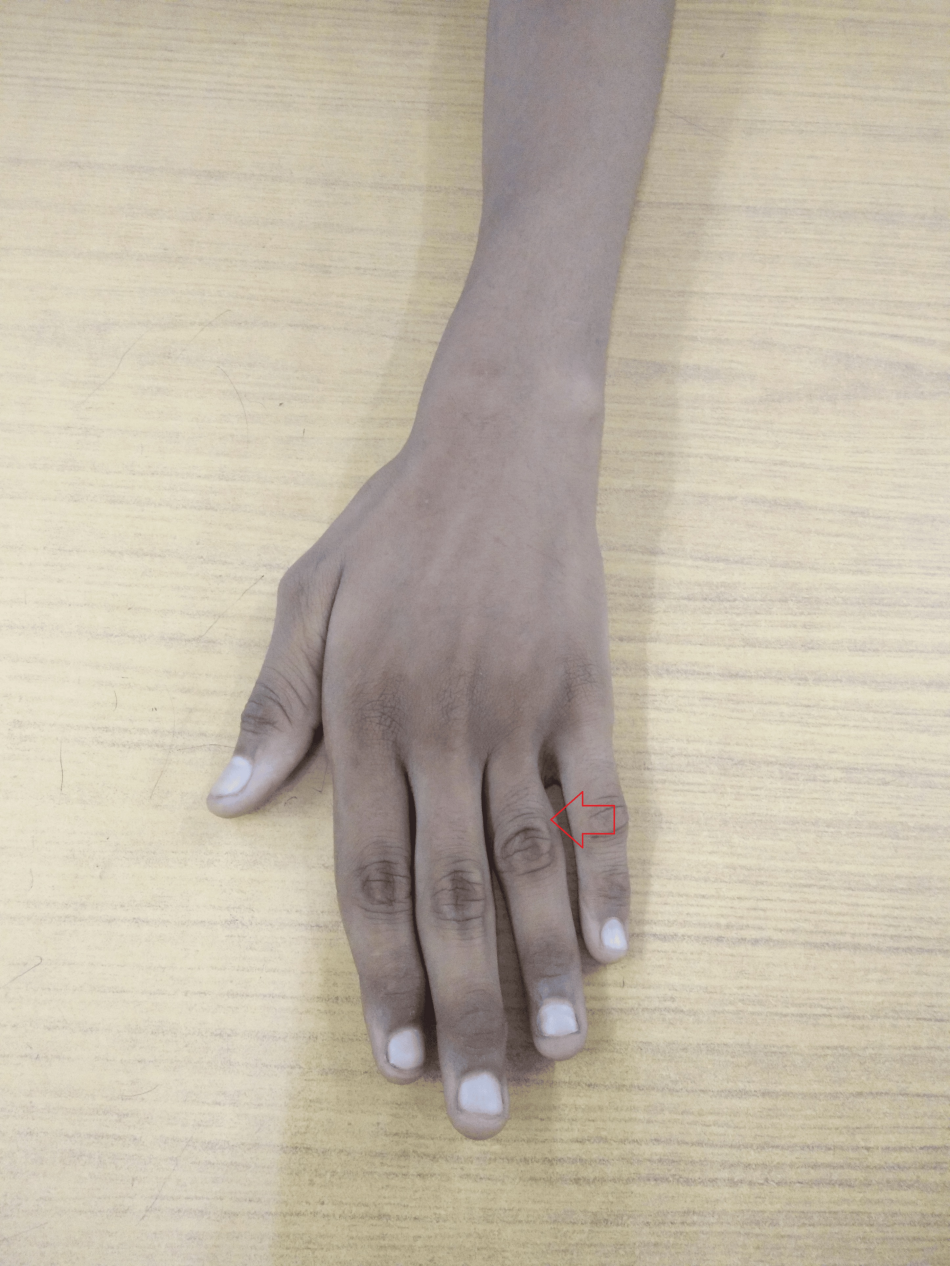
Gross image of the left ring finger showing no swelling.

**Figure 7:**
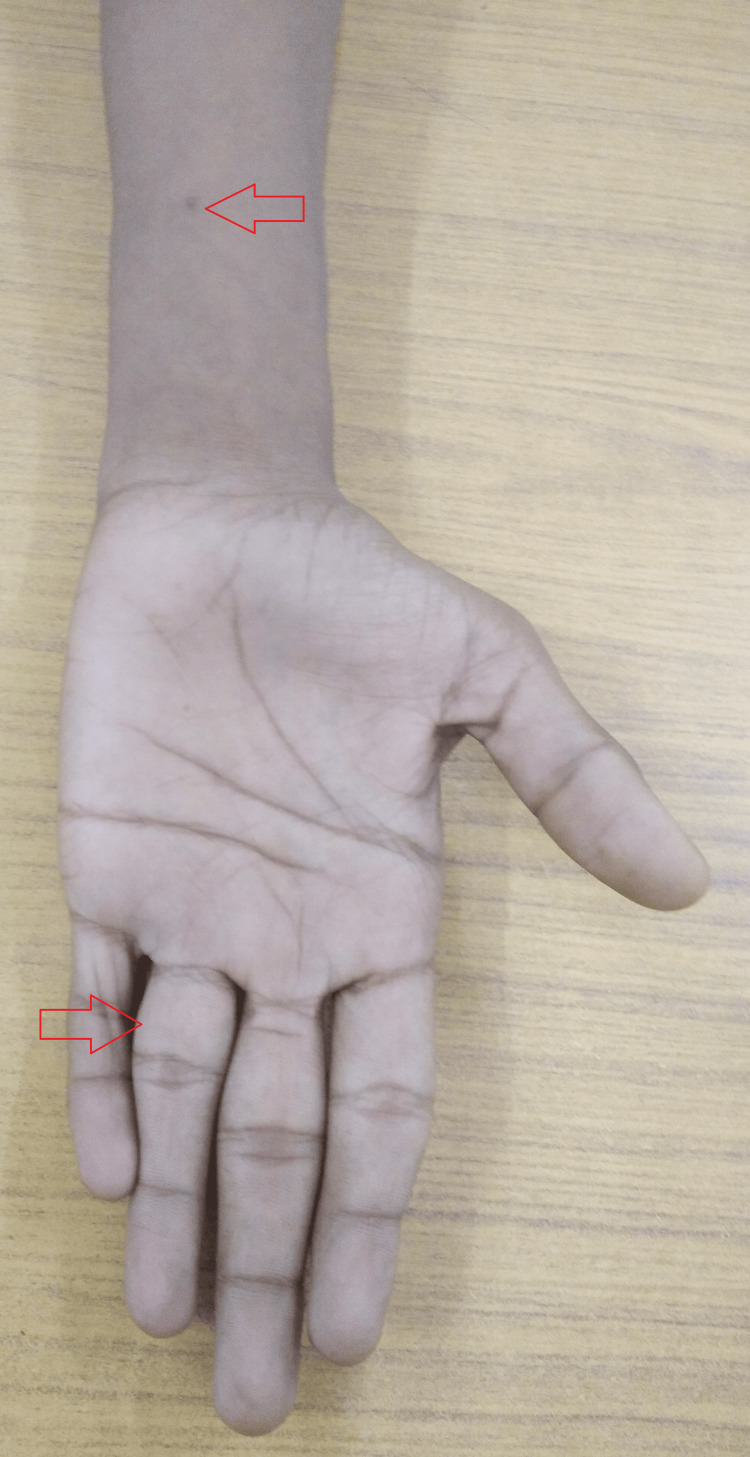
Gross image of the left ring finger and distal forearm.

Along with dietary counseling, he was advised regular follow-up in the outpatient department and treatment adherence. At the completion of the intensive phase, there was a fair clinical improvement with a reduction in the size of the swellings and closure of the discharging sinus, and at the end of the nine-month follow-up, there was a remarkable clinical improvement with a reduction in the size of swelling at both the left ring finger and left forearm (Figures 6-7). Furthermore, he was counseled to complete his full course of anti-tuberculous drugs.

## Discussion

Spina ventosa, or tuberculous dactylitis, is a rare clinical condition. Boyer first identified spina ventosa, also known as tubercular dactylitis, in 1803, and Nelaton demonstrated its tuberculous origin in 1837 [6]. Rankin subsequently characterized it histologically in 1886, while Feilchenfeld first identified it radiographically in children in 1896 [5].

Moreover, when this condition is reported beyond six years of age, it is extremely rare and is reported in less than 15% of cases [7]. Spina ventosa is a term used to describe any bone lesion characterized by subperiosteal swelling and cortical bone destruction around the medullary canal, leading to radiographically identified bone enlargement and destruction [6]. It is present in approximately 2-4% of all cases of skeletal tuberculosis [5], with the proximal phalanx of the index and middle fingers being the most commonly affected bone [8]. 

It usually manifests as a painless swelling of a finger that lasts for a few months. Unlike acute osteomyelitis, it frequently has a benign course without pyrexia and acute inflammatory symptoms. A low-grade temperature and soreness at the affected location are possible. It might also be connected to the development of sinuses, as seen in the present case. Clinical manifestations of anorexia and weight loss are common [2].

A bacterial infection and the development of an abscess in the skeletal muscle are referred to as pyomyositis. The exact cause of pyomyositis is yet unknown. Pyomyositis, a rare condition in immunocompetent patients, is caused by an unusual infectious agent known as *Mycobacterium tuberculosis*. Usually, invasion from nearby structures causes tuberculous pyomyositis instead of primary infection, lymphatic dissemination, or hematogenous seeding [3].

Diagnostic delays are common in tuberculosis patients since most present with a missed diagnosis, with no evidence of active pulmonary tuberculosis in >50% of patients and no constitutional symptoms [1,5]. The disease's paucibacillary nature, clinical symptoms that mimic those of other musculoskeletal disorders, and hazy multimodal imaging presentations are other contributing factors. The initial signs are often soft tissue edema and periostitis, which lead to expansile bone disintegration and sequestrum formation [7].

The preferred imaging method for tuberculous dactylitis is standard X-ray. Typical radiographic findings include periostitis and fusiform soft tissue swelling. "Spina ventosa," (meaning "wind-filled sail") characterized by a cyst-like cavity forming from bone destruction ballooning out, is a distinctive feature. Differential diagnosis from pyogenic infections involves identifying widespread osteopenia and the absence of sequestration. Computed tomography scans reveal bone sclerosis and destruction, while magnetic resonance imaging is preferred for early detection of marrow and soft tissue involvement. Cytopathology (bone or synovium biopsy, fine needle aspiration cytology) excludes other disorders. The positive culture of *Mycobacterium tuberculosis *from bone tissue is still the gold standard for diagnosing osseous tuberculosis [2]. However, the availability of tests like the cartridge-based nucleic acid amplification test that provides quick results with rifampicin resistance is advised per the national and WHO guidelines [9].

Management is done mainly by anti-tubercular drugs [1,5]. As advised by the Indian national standards or the guidelines, the prognosis is typically favorable, and the course of treatment essentially lasts for 12 months. The evaluation of the patient's condition at the end of a year establishes the next steps in the treatment plan [9]. In certain circumstances, surgical treatments are recommended for both diagnosis and treatment (e.g., carpal tunnel syndrome, abscess drainage, synovial excision, etc.) [4].

## Conclusions

An exceedingly rare case of spina ventosa of the left ring finger with tubercular pyomyositis in the forearm muscles in a 15-year-old Indian male with no pulmonary seeding is presented. The diagnosis was challenging, as even in endemic settings, such conditions are rare. The situation becomes more difficult as spina ventosa beyond six years is seldom reported, and pyomyositis is the least common site of extraspinal musculoskeletal tuberculosis. However, this case was timely diagnosed, and the same was evident by a clinical improvement at 12 months of the treatment. This case also stresses the need for documenting such cases to disseminate information about rare presentations of common diseases for timely diagnosis and management, as this could compromise the treatment outcomes. Moreover, this case also emphasizes the need for nationwide free advanced radiometric investigations, which would help in timely diagnosis.
